# Wellness, Mood, Sleep, and Performance in a Women's National Basketball Team during International Competition

**DOI:** 10.5114/jhk/200117

**Published:** 2025-04-30

**Authors:** Stephen P. Bird, Renée L. Parsons-Smith, Rachel King, Peter C. Terry

**Affiliations:** 1 School of Health and Medical Sciences, University of Southern Queensland, Ipswich, Australia.; 2 School of Psychology and Wellbeing, University of Southern Queensland, Ipswich, Australia.; 3 School of Mathematics, Physics and Computing, University of Southern Queensland, Toowoomba, Australia.; 4 Centre for Health Research, University of Southern Queensland, Toowoomba, Australia.

**Keywords:** team sports, mental health, female athletes, stress

## Abstract

The purposes of this study were to examine relationships between athlete self-report measures of wellness, mood, sleep, and performance indicators of a women's national basketball team during international competition (2023 FIBA Women’s Asia Cup). Physical and psychological wellness were recorded using a cloud-based athlete management system, mood was assessed using the 24-item Brunel Mood Scale (BRUMS), and performance was evaluated by player self-assessment and the National Basketball Association’s (NBA) player efficiency score. Wellness indicators were rated at or either side of a normal rating (i.e., 3 on a 1–5 scale), as was self-rated performance. Mean sleep duration was 8:13 hours (± 1:13), with large individual differences in retiring time and sleep duration. Mood scores showed a general pattern of declining tension and vigour over the 14-day assessment period. Mood subscales predicted 49.0% of variance in wellbeing scores, 46.8% of variance in preparedness scores, and 26.0% of variance in player efficiency scores. Wellbeing indicators predicted 34.9% of self-rated performance variance and mood subscale scores predicted 27.7% of variance. These findings suggest that female athletes may be particularly susceptible to mood disturbances impacting performance and highlight the potential for targeted interventions, including stress management and sleep optimization, to mitigate these stressors and support mental wellbeing. These strategies may enhance both psychological health and athletic performance during the high stress demands associated with international competition.

## Introduction

There is growing importance of the wellness of athletes in elite sport whilst also working to maximize performance (Edwards et al., 2018; Saw et al., 2016). To that end, monitoring athlete self-reported measures (ASRM), such as muscle soreness, fatigue, sleep quality, stress, and mood on a regular basis to evaluate wellness and accompanying training load tolerance (Saw et al., 2015a) has become commonplace (Duignan et al., 2020; Saw et al., 2015b, 2016). In a survey of Australian and New Zealand high-performance sport programs, Taylor and colleagues (2012) identified ASRM as the most commonly and frequently used fatigue-monitoring tools, with 84% of respondents assessing overall athlete wellness via this method as part of the athlete monitoring process. While debate exists regarding the validity of the single-item ASRM (Duignan et al., 2020; Saw et al., 2016), it could be argued that single-item ASRM are less monotonous and time-consuming, and thus may lead to increased relevance and greater survey compliance, especially during intensified training periods and pinnacle sporting events (i.e., Olympic Games, World Championships). Furthermore, custom single-item ASRM may allow performance staff to identify relevant ASRM domains within a sport-specific context, which is essential to create organizational buy-in during implementation of the monitoring process (Saw et al., 2015a).

Sport-specific manifestations of mental fatigue are regarded as a psychobiological state, caused by prolonged periods of extremely demanding and focused cognitive activity (Marcora et al., 2009), which may exacerbate a negative stress response. Elite athletes experiencing mental fatigue report a variety of symptoms such as feelings of disengagement, decreased motivation and enthusiasm, increased displays of emotion and withdrawal, reduced concentration, and decreased discipline and attention to detail (Russell et al., 2022). Not surprisingly, elite athletes are constantly exposed to stressors that may impair mental health and negatively impact sleep quality. Poor sleep is often reported in elite athletes, especially in the nights prior to competition. Biggins and colleagues (2021) reported that nearly a quarter of elite athletes experienced moderate to severe sleep problems at international competition. Mason et al. (2022) reported similar findings in 40 elite international track and field athletes, of whom 50% were female, from six different countries. Poor sleep is not only linked to mental fatigue (Andrade et al., 2019; Cook and Charest, 2023), but also risks to mental health (Terry and Parsons-Smith, 2021). Recently, Moen and colleagues (2021) reported bidirectional relationships between sleep and perceived fatigue in elite female soccer players, which might impair physical performance. Collectively, monitoring relevant sleep indices and implementing strategies to help prevent the onset of mental fatigue are crucial in safeguarding the wellness of elite athletes.

Over several decades, regular mood assessment has been shown to predict both psychological wellness and the performance outcome (Beedie et al., 2000; Morgan et al., 1987). Morgan’s mental health model (Morgan et al., 1987) postulated that a mood profile referred to as the iceberg profile, which is characterized by above average scores for vigor combined with below average scores for tension, depression, anger, fatigue, and confusion, is indicative of positive mental health and is associated with superior sports performance. Subsequent meta-analytic reviews of the mood and sport performance literature have provided support for the benefits of the iceberg mood profile (Beedie et al., 2000). Several other mood profiles have been identified in the sport psychology literature. For example, the inverse iceberg profile, characterized by above average scores for tension, depression, anger, fatigue, and confusion, coupled with below average scores for vigor, has been linked to overtraining syndrome (Morgan et al., 1987), poor athletic performance (Beedie et al., 2000) and a heightened risk of mental health issues (Terry and Parsons-Smith, 2021). Another mood profile that is indicative of poor performance and elevated risk of psychopathology, referred to as the inverse Everest profile, is characterized by low vigor scores, high scores for tension and fatigue, and very high scores for depression, anger, and confusion (Parsons-Smith et al., 2017). The surface profile is characterized by average scores on all mood dimensions, the submerged profile by below average scores on all mood dimensions, and the shark fin profile by below average scores for tension, depression, anger, vigor, and confusion, combined with very high fatigue scores (Parsons-Smith et al., 2017).

In a pre-cursor to the present study, an investigation by Lane and Chappell (2001), which explored the relationship between pre-game mood and individual performance among 10 members of the Great Britain men’s basketball team at the 1997 World Student Games, found that predicting performance from mood scores was highly individualized. Five players showed significant relationships between mood and performance, whereas the remaining five showed no relationship. Overall, mood accounted for 9% of performance variance, but up to 40% of performance variance among some players. Game performance has been described as a ‘function of the differences in the final score of the game’ (García et al., 2013). Collectively, it is essential to consider variables that may influence individual player readiness and impact game performance. Therefore, the aim of the present study was to investigate wellness, mood, sleep, and performance indicators of a senior women’s national basketball team during international competition.

## Methods

### Participants

Participants were 12 elite female basketball players from the New Zealand women’s senior national team competing at the 2023 International Basketball Federation (FIBA) Women’s Asia Cup. Player ages ranged from 18 to 29 years (M = 23.7 ± 3.3 years) and the number of international games played prior to the tournament ranged from 2 to 60 (M = 23.3 ± 21.5 games).

### Measures

#### Measurement of Wellness and Sleep

Athlete self-report measures (ASRM) of wellness were collected daily during the investigation period via a cloud-based athlete management system (VisualCoaching Pro [VCP], Melbourne, Australia). The ASRM used were perceptions of sleep quality, readiness, recovery, feelings, energy, muscle soreness, and leg heaviness and were reported on a 5-point Likert scale (scores of 1 to 5), where lower numbers indicated a negative response, and higher numbers indicated a positive response. ASRM have been reported to be sensitive to changes in athlete health and indicators of wellness among elite athletes (Saw et al., 2016). Overall wellness was calculated by summing scores for sleep quality, feelings, and energy, and overall preparedness by summing scores for sleep quality, recovery, and energy. Night retiring time (lights out each night to go to sleep) and sleep quantity (hours slept) were also self-reported with players hosted in twin-share hotel rooms. These measures (collectively referred to as ASRM) relate to the female athlete health domains, as per the International Olympic Committee consensus statement (Moore et al., 2023). The team physician (on-site) and sport psychology provider (remote) were available for follow-up consultation to provide support to players experiencing negative wellness indicators.

#### Measurement of Mood

Mood was assessed using the Brunel Mood Scale (Terry et al., 2003), a 24-item scale to assess the mood dimensions of tension, depression, anger, vigor, fatigue, and confusion (4 items each). Players indicated how they were feeling *“right now”* on a 5-point scale; 0 = not at all, 1 = a little, 2 = moderately, 3 = quite a bit, and 4 = extremely. Subscale scores ranged from 0 to 16, with higher scores indicating higher levels of a mood dimension. The BRUMS has demonstrated robust psychometric characteristics (Lane et al., 2007; Terry et al., 2003) and has been globally used to assess mood in athletic populations (Lane and Chappell, 2001; Terry and Parsons-Smith, 2021). Players completed the BRUMS on nine occasions during the 14-day investigation period.

#### Measurement of Game Performance

Two game performance measures were used. The first performance measure was a *Self-rating of Performance* by each player after every game on a 5-point Likert scale, where higher numbers indicated better self-rated performance. Publicly available box-score data (International Basketball Federation, 2023) provided game-related statistics required to determine the second performance measure, termed the *Individual Player Efficiency Score*. This was calculated using the *Player Efficiency Rating* (PER), as per Hollinger (2007), which determines the rating of a player’s performance, according to the following formula:

#### Efficiency (EFF) = (PTS + REB + AST + STL + BLK – Missed FG – Missed FT – TO)

where:

PTS = number of points scored by the player

REB = number of rebounds by the player

AST = number of assists by the player

STL = number of steals by the player

BLK = number of blocks by the player

Missed FG = number of field goals missed by the player

Missed FT = number of free throws missed by the player

TO = number of turnovers the player has allowed

### Design and Procedures

Prior to data collection, the lead author attended a national training camp with the team, coaching and support staff during which the purpose of the research, procedures and possible risks were explained. Participants were familiar with all procedures which were consistent with national team protocols and were made aware that (1) their participation was voluntary; (2) they could withdraw at any time; and (3) completion of the study questionnaire(s) constituted their consent to participate. The study was conducted in accordance with the Declaration of Helsinki and approved by the Human Research Ethics Committee of the University of Southern Queensland (protocol code: #H21REA182; approval date: 01 September 2021).

**Figure 1:**
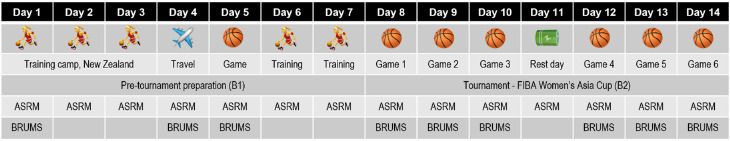
Schematic of the data collection timeline. B1, Block 1 (Pre-tournament preparation); B2, Block 2 (Tournament).

Data collection occurred over a 14-day period that encompassed a national training camp, plus travelling to and competing in the 2023 FIBA Women’s Asia Cup tournament (Sydney, Australia). This period was divided into two 7-day blocks (B1, pre-tournament preparation and B2, tournament). B1 covered a 3-day national training camp, international travel from New Zealand to Australia, and one pre-tournament game. B2 covered the Asia Cup tournament, with six games played in seven days. Training times were scheduled between 10:00–12:00 pm and/or 4:00–6:00 pm, with these times generally consistent during B2. Game tip-off times were as follows: Games 1 and 3, 11:00 am; Games 2, 4 and 5, 5:00 pm; and Game 6, 12:30 pm. The lead author attended the competition as Performance Team Lead, which allowed for direct communication with athletes and facilitated administration of the study questionnaires. Participants provided wellness and mood data electronically (via a cloud-based athlete management system) during the prescribed period of 07:00–09:00 am on the days identified in Figure 1, with the time required to complete data entry less than 10 min.

### Statistical Analysis

Data were collated for analysis using SPSS Version 28 (IBM Corp, Armonk, NY) and descriptive statistics were calculated for all variables. To assist the interpretation of group and individual mood profiles, all mood subscale scores were converted into standard scores (T-scores) using tables of normative data for female athletes (Terry and Parsons-Smith, 2021). Pearson correlations were used to quantify relationships among indicators of wellness, mood subscale scores, and performance measures. Multivariate analysis of variance (MANOVA) was used to compare mood scores across blocks, and effect sizes (Cohen’s *d*) were calculated. Multiple regression analysis was used to determine if variance in individual performance could be predicted from wellness and/or mood scores. The alpha level was set at *p* < 0.05 for all analyses, except for the univariate follow-up tests for MANOVA, where a Bonferroni adjustment was made to guard against a Type I error, resulting in an adjusted alpha level of *p* < 0.008. Correlations and effect sizes were interpreted in line with the recommendations of Cohen (1992), where a correlation coefficient (*r*) of 0.10, 0.30, and 0.50, was indicative of small, medium, and large effects, respectively, and an effect size (*d*) of 0.20, 0.50, and 0.80, was indicative of small, medium, and large effects, respectively.

## Results

Table 1 shows the total number of observations captured from the measures of wellness, BRUMS, and performance. Analyses were conducted at both a group level and an individual level.

**Table 1:** Data capture during the investigation period.

Measure	Items	Days	Submissions	Data points
Wellness (ASRM)	8	14	168	1,344
Mood (BRUMS)	24	9	99	2,376
Self-rated performance	1	6	6	6
Efficiency score	8	6	6	48

### Wellness

**Figure 2:**
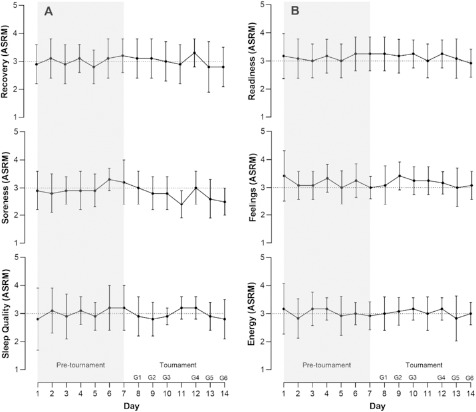
Mean (SD) for wellness and sleep across the 14-day investigation period during the 2023 FIBA Women’s Asia Cup. (A) Sleep quality, Soreness, and Recovery; (B) Energy, Feelings and Readiness. *ASRM, athlete self-report measure; G1, pool game 1; G2, pool game 2; G3, pool game 3; G4, quarter-final; G5, semi-final; G6, bronze medal playoff*

Mean wellness indicators for the 12 team members on each day are shown in Figure 2. All indicators were rated on a 1–5 scale (except for hours slept). It is apparent that most indicators were rated at or either side of a ‘normal’ rating of 3. This also applied to the performance self-rating (during training or game).

### Sleep

**Figure 3:**
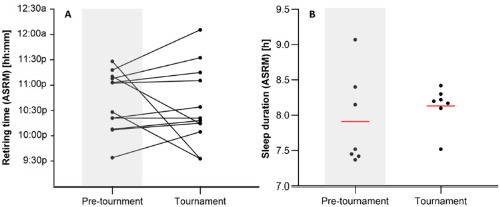
Athlete self-reported mean night retiring time (A) and mean sleep duration (B) during pre-tournament preparation period and tournament. Line in figure B represents the mean.

Figure 3 shows the mean night retiring times for the 12 athletes (A) and mean sleep duration (B) over each 7-day block during the pre-tournament preparation and during the tournament. Retiring times demonstrated a high level of within- and between-player variability. Pre-tournament and tournament night mean retiring times were 11:13 pm (± 2:19 h) and 11:09 pm (± 2:03 h), respectively. It is notable that most of the team (10 players) were typically in bed prior to midnight during the tournament. Mean sleep duration during the pre-tournament preparation and tournament was 8:07 h (± 1:21) and 8:19 h (± 1:05), respectively. Notably, mean sleep duration demonstrated a cluster during the tournament (Figure 3B). Mean sleep duration ranged from 7:37 h (± 0:49) on day 5 to 9:02 h (± 1:47) on day 6, with an overall mean of 8:13 h (± 1:13). On 15 occasions sleep duration was at or below the set minimum sleep threshold of six hours, with 11 of these occurring during the pre-tournament period.

Correlations among wellness indicators are shown in Table 2. Significant positive relationships were shown among sleep duration, sleep quality, and recovery, and also among recovery, readiness, soreness, and leg heaviness. Feelings were positively correlated with energy. Wellness indicators were significantly correlated with self-rated performance. It should be noted that self-rated performance included overall daily performance in training and/or games.

**Table 2:** Correlations among wellness indicators for 12 team members (N = 168).

ASRM	Sleep Quality		Sleep (hrs)	Readiness	Recovery	Feelings	Energy	Soreness	Leg Heaviness
Sleep (hrs)		0.55^†^							
Readiness		0.54^†^	0.38^†^						
Recovery		0.48^†^	0.29^†^	0.73^†^					
Feelings		0.47^†^	0.39^†^	0.61^†^	0.54^†^				
Energy		0.46^†^	0.34^†^	0.62^†^	0.57^†^	0.73^†^			
Soreness		0.25^†^	0.08	0.51^†^	0.54^†^	0.15	0.26^†^		
Leg Heaviness		0.14	0.05	0.40^†^	0.46^†^	0.11	0.21*	0.69^†^	
Performance		0.30^†^	0.21*	0.53^†^	0.40^†^	0.43^†^	0.49^†^	0.22*	0.29^†^

Abbreviations. ASRM = Athlete self-reported measure. Note. Self-rated performance included overall daily performance in training and/or games; ^†^ p < 0.001, * p < 0.01

### Mood

**Figure 4:**
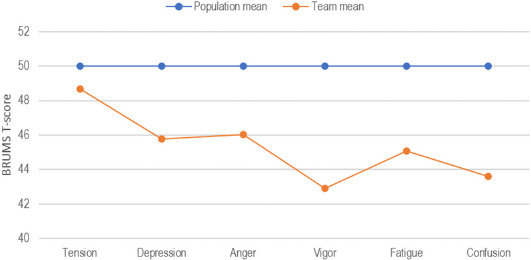
Mean mood scores (N = 99) plotted against population norms for female athletes (N = 877) (Terry and Parsons-Smith, 2021).

Mean scores for the whole 14-day period (i.e., B1 and B2 combined) were significantly lower than population norms for female athletes (Terry and Parsons-Smith, 2021) on all subscales except tension (tension: *t* = 1.29, *p* > 0.05; depression: *t* = 4.05, *p* < 0.001; anger: *t* = 4.03, *p* < 0.001; vigor: *t* = 6.80, *p* < 0.001; fatigue: *t* = 4.73, *p* < 0.001; confusion: *t* = 6.15, *p* < 0.001). This profile is shown in Figure 4 and is referred to as a submerged mood profile (Parsons-Smith et al., 2017) as all subscale scores are below the population mean, which is metaphorically regarded as the water line. Frequency distributions showed a very large proportion of zero scores for some mood subscales, in particular for anger (97.3%) and confusion (90.8%).

MANOVA showed a significant multivariate difference in mood scores between B1 and B2 (Wilks’ *λ* = 0.841, *F*_6,92_ = 2.89, *p* = 0.01), with higher scores for tension, vigor, and fatigue reported during the preparation period than the tournament period, and almost identical scores for depression, anger, and confusion across the two blocks of time. Applying a Bonferroni adjustment to the alpha level for univariate analyses (0.05/6 = 0.008), none of the differences in subscale scores reached statistical significance, although Cohen’s *d* showed differences to be small-to-moderate in magnitude for vigor, and moderate for tension and fatigue.

**Figure 5:**
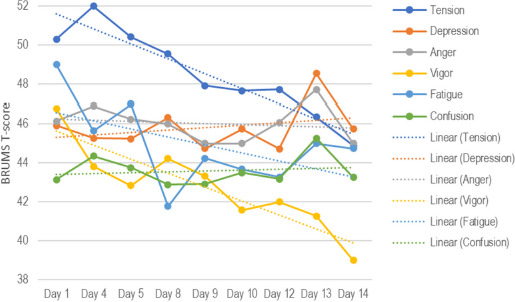
Mood changes over the 14-day investigation period.

Figure 5 shows changes in team mood scores over time. Trendlines showed a steady reduction over time for tension and vigor, and to a lesser extent fatigue, whereas scores for depression, anger, and confusion remained steady except for a marked increase on Day 13, the day of the semi-final game. The observed fluctuations did not represent statistically significant changes.

### Prediction of Overall Wellness and Preparedness from Mood

Multiple linear regression showed that, collectively, the six mood subscales scores (tension, depression, anger, vigor, fatigue, confusion) predicted 49.0% of the variance in wellness scores (*F*_6,92_ = 14.74, *p* < 0.001, *r*^2^ = 0.49). Lower depression scores (*β* = −1.12 *p* < 0.001), lower confusion scores (*β* = –1.28 *p* < 0.001), and higher vigor scores (*β* = 0.54, *p* < 0.001) were all significant predictors of higher wellness scores. Multiple linear regression also showed that the mood subscales scores collectively predicted 46.8% of the variance in preparedness scores (*F*_6,92_ = 13.51, *p* < 0.001, *r*^2^ = 0.47). Lower depression scores (*β* = –1.08 *p* < 0.001), lower confusion scores (β = –1.30 *p* < 0.001), lower fatigue scores (β = –0.41 *p* < 0.001), and higher vigor scores (β = 0.41, *p* < 0.001) were all significant predictors of higher preparedness scores.

### Performance

Overall, the team won three games (vs. Korea, Lebanon, Philippines) and lost three games (vs. China, Japan, Australia) to finish 4^th^ in the eight team tournament, despite being ranked 5^th^ in this tournamant. Their best performance was a 66-64 victory in Game 1 over a team ranked 17 spots above them in the FIBA world rankings. As a result of their performances in the tournament, New Zealand qualified for the final 16 team 2024 FIBA Women's Olympic Qualification Tournament and had one player selected to the tournament All-Star Five.

Efficiency scores across all team members ranged from –2 to 32 (M = 6.4 ± 8.1). Multiple linear regression showed that, collectively, the six mood subscales scores (tension, depression, anger, vigor, fatigue, confusion) predicted 26.0% of the variance in efficiency scores (F_6,48_ = 2.81, *p* = 0.02, *r*^2^ = 0.26). Higher tension scores (*β* = 0.78, *p* = 0.002), lower fatigue scores (β = –0.51, *p* = 0.016), and lower vigor scores (β = –0.36, *p* = 0.016) were all significant predictors of higher efficiency scores. None of the wellness indicators were significant predictors of efficiency scores.

In terms of self-rated performance, mood subscale scores collectively predicted 27.7% of the variance in performance (*F_6,92_* = 5.86, *p* < 0.001, *r^2^* = 0.277). Low depression scores (*β* = –0.82, *p* < 0.001), high vigor scores (*β* = 0.25, *p* = 0.011), and low confusion scores (*β* = –1.19, *p* = 0.001) were all significant predictors of self-rated performance. Wellness indicators collectively predicted 34.9% of the variance in self-rated performance (*F_8,159_* = 10.63, *p* < 0.001, *r^2^* = 0.349). Higher readiness (*β* = 0.40, *p* < 0.001), higher energy (*β* = 0.24, *p* = 0.017), and lower leg heaviness (*β* = 0.22, *p* = 0.015) ratings were all significant predictors of better self-rated performance. There was no relationship between self-rated performance and the objective performance measure in the form of player efficiency scores (*r* = –0.06, *p* = 0.66).

## Discussion

To our knowledge this is the first study to adopt an interdisciplinary approach, bringing together researchers with expertise in strength and conditioning, sport science, sport psychology, and mental health, to examine wellness, mood, sleep, and performance indicators among an elite women’s basketball team competing in a major international tournament.

A prominent feature of the mood scores was the very low scores reported by team members, significantly lower than normative scores for female athletes (Terry and Parsons-Smith, 2021). Below average pregame scores for tension, depression, anger, fatigue, and confusion have typically been associated with successful performances in sport (Beedie et al., 2000). Furthermore, the submerged profile reported by the team has been linked to successful performance in sports that put a premium on remaining calm and unemotional, such as shooting and archery (Li et al., 2021). However, although the sport of basketball requires extreme calmness at times, such as when preparing to shoot a free throw, the dynamic nature of the sport may require higher levels of vigor than reported by the participants in our study. The mean raw score for vigor prior to the six games of the tournament was 5.06 on a scale of 0−16. The four items in the vigor scale are lively, energetic, active, and alert; a per item mean of 1.27. This corresponds to the players feeling, on average, only a little lively, energetic, active, and alert, which is unlikely to be an optimal mindset for basketball performancee. Research into mood regulation strategies among athletes (Terry et al., 2006) showed the most popular and effective strategies to increase feelings of vigor on the day of a competition were engaging in physical pre-competition activities, listening to fast, upbeat music, using sport-related imagery, focusing on competition strategies, and using humor. Our results suggest there is a case for providing elite basketball players with pre-game performance arousal strategies for regulating mood.

The group mean scores in the present study share similarities with those reported by Lane and Chappell (2001). In their study, group mean scores for tension, depression, anger, fatigue, and confusion were similarly below population means (43^rd^ to 46^th^ percentile) whereas mean vigor scores were above the 50^th^ percentile, reflecting an iceberg profile, generally associated with good athletic performance (Beedie et al., 2000). In the present study, another clear feature of the group mood scores was the steady reduction over time of tension and vigor, and to a lesser extent fatigue. Depression, anger, and confusion scores remained steady throughout the tournament, except for higher scores on the day of the semi-final game (vs. Japan), a team ranked 20 places higher in the FIBA World Rankings. Some of these mood trends could be interpreted as sub-optimal, in that a pattern of maintaining or even increasing vigor over the course of the tournament whilst avoiding increases in the other mood dimensions may be advantageous. However, direct comparisons between genders are challenging due to potential differences related to biopsychosocial factors.

In terms of predicting performance, mood scores, in particular tension, fatigue, and vigor scores, predicted statistically significant 26.0% of the variance in player efficiency scores, whereas wellbeing indicators did not significantly predict player efficiency. This level of predictive effectiveness was higher than previously reported (Lane and Chappell, 2001), where mood scores predicted just 9% of overall performance variance using a self-rating of performance. Mood scores, especially for depression, vigor, and confusion, predicted 27.7% of the variance in self-rated performance. However, contrary to a recent meta-analysis by Lochbaum et al. (2023), self-rated performance was a relatively insensitive indicator, given that the team’s best performance (Game 1) was rated lower than a poorer performance in Game 2 and was unrelated to objective performance. The more objective efficiency scores, which are used as a measure of basketball performance (Hollinger, 2007; International Basketball Federation, 2023), proved to be a more sensitive indicator of performance. This highlights the significant challenge of identifying suitable measures of individual performance within team sports. Some of the specific overall mood-performance relationships ran counter to previous findings. For example, higher tension scores and lower vigor scores were associated with better objective performance, whereas the opposite is typically the case (Beedie et al., 2000; Lane and Chappell, 2001). These results may possibly be anomalous due to the small number of games played or they may reflect idiosyncratic effects of mood on performance for the participants involved.

Sleep is recognized as a critical factor to optimize athlete health, recovery, and performance (Bird, 2013; Singh et al., 2021). However, sleep disturbances prior to and during competition are highly prevalent among athletes (Brauer, 2022), and may negatively impact mood. An expert consensus (Walsh et al., 2021) highlights that elite athletes are particularly susceptible to sleep insufficiency, characterized by habitual short sleep duration (<7 h/night) and poor sleep quality (e.g., fragmented sleep). Such sleep disturbances can negatively impact athlete health and immune regulation, and result in increased daytime sleepiness, reduced physical readiness, poor cognitive processes (i.e., visual tracking, attention, and mood), and suboptimal recovery; all of which contribute to injury risk (Charest and Grandner, 2020). Importantly, the International Olympic Committee (IOC) mental health consensus statement (Reardon et al., 2019) refers to sleep as not only a major contributor to athletic performance, but as a fundamental feature of athlete mental health and considers sleep health in terms of sufficiency (i.e., at least 7 h/night for adults). However, elite athletes have greater sleep requirements given their exposure to heavy periods of training and competition, and increased travel demands.

In the current study, mean sleep duration was 8:07 ± 1:21 h during pre-tournament preparation compared to 8:19 ± 1:05 h during the tournament. In comparison to professional female players competing in the Australian Women’s National Basketball League (WNBL), this is similar mean sleep duration to that reported by Dunican and colleagues (2021). Notably, across the course of the 14-day investigation period five players were classified as habitual short sleepers, according to Walsh et al. (2021). This is a significant consideration in the context of the current study given the effect of poor sleep health on recovery and basketball performance (Mah et al., 2011; Ochoa Lacar et al., 2022; Singh et al., 2021). While several sport-specific and societal factors have been reported to contribute to sleep insufficiency in elite athletes (Brauer, 2022; Singh et al., 2021; Walsh et al., 2021), shared room accommodation during training camps and competition is a key consideration. Such sleep environments may place athletes at increased risk of poor sleep quality, sleep insufficiency and potential sleep deprivation. Sleeping in unfamiliar, shared room settings (i.e., athlete dorms or hotel rooms) can result in significant disruption to sleep routines as athletes adapt to a new sleep environment. This may expose athletes to several unaccustomed sleep environment factors, such as excessive levels of light and noise, changes in room temperature, unfavorable sleep surfaces (mattress and pillow quality), increased screen time, and communication (bedtime stories). Such factors may have contributed to the high within- and between-player variability reported in sleep duration and night retiring time in the current study. Furthermore, another consideration is game tip-off time. It is not uncommon for teams to arrive two hours before game tip-off. This will impact meal timing and sleep episodes. In this regard, the earliest game tip-off time was 11:00 am (Game 1 and 3 *)* with the latest at 5:00 pm (Game 2, 4, and 5 *)*. Athletes will schedule their morning sleep awaking time and napping awake time with this in mind.

Of major interest is the potential link between sleep insufficiency and mood factors. In a cross-sectional study of 1,041 elite athletes (35.5% female), Andrade et al. (2019) found that among the six mood factors, an increase in confusion, fatigue, and tension with a decrease in vigor was associated with poor sleep. This extends the earlier work of Lastella et al. (2014) who reported relationships among tension, sleep quality, and sleep disruptions in marathon runners. Poor sleep quality, sleep time, and waking at night were negatively correlated with precompetitive sleep quality. Our findings corroborate other studies that also report relationships between sleep quality and mood factors in elite athletes. Collectively, an increase in negative mood factors leads to worsening sleep quality whereas high vigor may contribute to better sleep quality, and vice versa. Sleep extension (Mah et al., 2011) and/or banking sleep (Rupp et al., 2009) strategies are often used to allow athletes to obtain increased sleep prior to periods of anticipated sleep loss. This approach involves scheduling longer sleep periods than normal, and usually consists of a *‘protected sleep window’* of 9–10 hours (Walsh et al., 2021). Athletes in the current investigation specifically employed sleep extension on days that did not involve an AM training session. Coupled with sleep hygiene education (Bird, 2013; Dunican et al., 2021), such strategies offer athletes a practical means to reduce sleep insufficiency associated with international competition and may have implications for the provision of sleep interventions.

When interpreting the findings, there are potential limitations which should be acknowledged. Firstly, the current study was a team-based case series design. A typical national basketball team competing at pinnacle international tournaments includes 12 or fewer players. As such, the small sample (*n* = 12) used in our study requires consideration. Future studies would benefit from including players from the wider squad depth chart to gather baseline data. Secondly, from a sleep perspective, the current study focused on self-reported sleep duration and sleep quality. Consequently, we cannot rule out that athletes may exaggerate their responses to meet expectations. Furthermore, Power et al. (2023) highlight that covariates, such as chronotype, menstrual cycle, and sleep hygiene may influence sleep health in female basketball players. While sleep hygiene education and recommendations (Bird, 2013; Dunican et al., 2021) were provided, we did not control specific environment factors known to affect sleep health (i.e., room temperature and humidity, naps, and electronic device use and screen time immediately before bedtime). Lastly, no baseline data were collected prior to entering the final national team training camp (Pre-tournament preparation; Day 1 to 7). Conducting ecological valid, real-world performance research on elite athletes presents unique challenges, with context-specific limitations. However, such research is highly valuable for examining the impact of intensified training and international competition on elite female basketball players and the implications for female athlete health and performance more generally.

## Conclusions

The present study investigated domains related to wellness, mood, sleep, and performance among elite female basketball players during international competition. Collectively, the current findings underscore the importance of implementing targeted strategies to enhance sleep, wellness, and positive mood among elite female basketball players, particularly during the high-stress period of international competition. Interventions such as stress management, sleep optimization techniques, emotional awareness and mental wellness support may be vital in mitigating competition-related stressors. This is a key consideration as female athletes often experience lower mental wellness compared to their male counterparts (Pascoe et al., 2022), with sub-clinical (undiagnosable) mental health symptoms such as anxiety, depression, and mood disturbances negatively impacting emotional wellbeing and performance. By addressing these factors, teams can help safeguard the mental health and overall performance of female athletes both before and during international competition.
